# Improving accessibility for outpatients in specialist clinics: reducing long waiting times and waiting lists with a simple analytic approach

**DOI:** 10.1186/s12913-018-3635-3

**Published:** 2018-11-01

**Authors:** Karl Arne Johannessen, Nina Alexandersen

**Affiliations:** 1 The Intervention Center, Oslo university hospital, Oslo, Norway; 2 Department of Health Management and Health Economics, University of Oslo, Oslo, Norway; 3 Norwegian Institute of Public Health, Oslo, Norway

**Keywords:** Waiting time, Quality improvement, LEAN, Outpatient booking, Planning horizon, Physician productivity, Norway

## Abstract

**Background:**

Lack of resources is often cited as a reason for long waiting times and queues in health services. However, recent research indicates these problems are related to factors such as uncoordinated variation of demand and capacity, planning horizons, and lower capacity than the potential of actual resources.

This study aimed to demonstrate that long waiting times and wait lists are not necessarily associated with increasing demand or changes in resources. We report how substantial reductions in waiting times/wait lists across a range of specialties was obtained by improvements of basic problems identified through value-stream mapping and unsophisticated analyses.

**Methods:**

In-depth analyses of current operational processes by value-stream mapping were used to identify bottlenecks and sources of waste. Waiting parameters and measures of demand and resources were assessed monthly from 12 months before the intervention to 6 months after the intervention. The effect of the intervention on reducing waiting time and number of patients waiting were evaluated by a difference-in-differences analysis.

**Results:**

Mean waiting time across all clinics was reduced from 162 + 69 days (range 74–312 days) at baseline to 52 + 10 days (range 41–74 days) 6 months after the intervention. The time needed to achieve a waiting time of 65 days varied from 4 to 21 months. The number of new patients waiting was reduced from 15,874 (range 369–2980) to 8922 (range 296–1650), and the number of delayed returning patients was reduced from 18,700 (310–3324) to 5993 (40–1337) (*p* < 0.01 for all). Improvement in waiting measures paralleled a significant increase in planning horizon.

**Conclusions:**

Significant improvements in accessibility for patients waiting for service may be achieved by applying unsophisticated methods and analyses and without increasing resources. Engagement of clinical management and involvement of front line personnel are important factors for improvement.

## Background

In many modern healthcare systems, patients may experience long waiting times (WT) for public health services. Constraining costs while providing adequate and timely access to services based on equity is challenging. Studies on whether political initiatives targeting long WT translate into improved access for patients have shown that success varies across policies [1–4].

There is substantial evidence that adding more resources will not necessarily improve accessibility and decrease queues, meaning understanding the mechanisms behind queuing is crucial [5–7]. If demand exceeds capacity, queues would increase indefinitely. A situation in which long WT are stable over time combined with steady demand and resources should trigger the hypothesis that resources are adequate and long WT are associated with organizational malfunctions. A study by Allder et al. from the National Health Service (NHS) suggested that wait lists often are relatively stable, that growing wait lists are unusual, and that a mismatch between the variation in demand and capacity often is a major cause of queuing [8]. Understanding the significance of an uncoordinated capacity and demand variation is important [2, 6]. Furthermore, reports from the NHS Institute and Norway show that patient admissions and completed consultations per consultant can vary by over 100% across hospitals in the same healthcare system [9–12]. This suggests that there would be substantial gain if lower-level performers could be improved to operate at the average level. Of note, actual capacity of a given resource may depend on the skill mix and how resources are organized [12–16].

In Norway, political initiatives to reduce WT include a legislated maximum waiting time guarantee (introduced in 1997 as a 6-month limit and gradually tightened), activity-based financing of hospitals (from 1997), and granting patients the right to free choice of hospital combined with removal of county border barriers (in 2001). Although some effects of these initiatives have been reported [1, 17], long WT did not improve in Norwegian hospitals until the last 3 years. Despite a near doubling of budgets in the last decade, the perception that long wait lists and WT are caused by deficit of resources has dominated the debate, with proponents insisting that Norway’s strong economy should trigger increased funding for hospitals.

However, despite the strong Norwegian economy, increasing resources as a sole solution is unsustainable, particularly given the health professional workforce deficit faced by most health systems; an imbalance complicated by changing population demographics and increasing demand for resources. Moreover, The Norwegian Institute for Studies of the Medical Profession has reported that only 50% of physician working time is spent with patients [18], suggesting it is important to identify suboptimal use of resources across health professional groups.

Numerous studies are available describing techniques that may be useful in this context. Two well-known specialized methods from industry which focuse on looking for waste, improving work flow and creating more value with less effort is the LEAN method (originating from the Toyota car manufacturing system) and Six Sigma (originating from Motorola). LEAN [19] has been applied in various healthcare settings and multiple clinical specialties [20–28]. Kullar et al. [29–33] used LEAN principles to improve efficiency, and Gijo et al. [32] applied Six Sigma to reduce WT [29–33]. However, several of these reports have discussed that implementing LEAN in clinical cultures may present other, more demanding challenges than implementing the model in industry. Whereas manufacturing companies seeking to adopt the “Toyota Method” often succeed with a continuous focus on solutions to improve processes, the necessary engagement of management and capability of continuously questioning existing solutions to solve identified problems by involving front-line personnel seems to be more challenging to implement among clinical leaders than other sectors [34–36].

Based on the current literature, the LEAN method remains disputed in clinical settings, and Moraros et al. [21] concluded that available evidence does not support the idea that LEAN leads to quality improvements in healthcare. Costa et al. [20] noted a need for more studies that show LEAN healthcare applications not limited to specific areas, along with studies from more countries as current LEAN reports are from a limited number of countries (mainly the US and UK). Daultani [37] concluded that there are knowledge gaps in determining the path a general or multi-specialty hospital should follow in implementing LEAN, and that there is limited research to guide managers in choosing an appropriate portfolio of LEAN projects within a given time frame. Furthermore, Radnor and Walley [23] warned against an implementation approach focused solely on LEAN tools, and noted that the majority of academic literature on LEAN in the public sector is descriptive and developmental in nature. Although there is increasing literature regarding LEAN in healthcare, synthesizing these reports to make evidence based knowledge is rather challenging as pointed out by Rotter et al. [38].

Our team has extensive experience of LEAN work in a variety of settings and we derived our methods in the current project from LEAN. However, as our primary goal was to reduce WT that breached the legal threshold within a fairly short period, we were not able to engage in improvements across entire organizations, and did not focus on in debt learning of LEAN as a method. We therefore chose not to aim for a full LEAN methodology, but rather focus on targeted actions, adapted to each clinic but based on some common principles.

### Background for the project

The Regional Health Authority South East, covering approximately 50% of Norwegian hospital services, addressed persisting long WT in 2014 through intensified efforts aiming to achieve the national standard average WT of 65 days in all hospitals. One initiative was launching a task force to assist selected clinics with the most extensive problems. This paper reports on changes in wait lists and WT following the work of this task force in 12 different outpatient clinics from four hospitals.

### Aim and scope of this paper

The project of this paper had a two-fold aim. First, we examined the association between long WT, demand, and resources in the 12 months before the intervention to build knowledge in this area. Second, we explored how application of targeted approaches, learned from LEAN principles, combined with unsophisticated Microsoft Excel analyses may affect long WT and wait lists across different medical specialties and clinics.

## Methods

Participating clinics were chosen at the discretion of the Health Authority South East, based on those with the longest WT or wait lists in their specialty in the region. Of 17 selected clinics, one chose to discontinue the process, and it was not possible to assess resource measures in four clinics because these data were integrated in larger units. Therefore, we included 12 clinics specialized in cardiology, pulmonology, gastroenterology, gastro-surgery, orthopedics, ear-nose- throat, ophthalmology, and neurology from four hospitals that could provide access to data for a 6-month follow-up period after the project ended.

During our project period, there was a general focus in all hospitals on improving accessibility. We therefore used a control group of 26 other clinics in the four hospitals that had WT > 65 days but where we did not intervene. These were used to compare our results for WT and PW in the period.

### Data sources

Reports of Norwegian wait lists and WT are published regularly on an open, publicly accessible platform [39]. Providing such information to stakeholders is useful to assess status and identify possible tradeoffs or inadequacies in responses to primary political goals. For example, there is concern that government-set priorities and targets to minimize time to start of treatment have triggered system behavior resulting in lower prioritization of returning patients (RP) who need follow-up [7, 8, 40, 41].

Measures of WT vary among OECD countries [4]. Examples of measures used in many national datasets are inpatient WT (from addition to the specialist list to treatment), referral-to-treatment WT (from referral by a GP/family doctor to treatment), WT for all patients on the list, and numbers on wait lists. These parameters are usually assessed by mean or median values, WT distribution across percentiles, and the number of patients waiting more than a specified threshold (e.g., 3–6 months in most systems). We used three time-related variables: evaluation time, measured as the time from GP referral of a new patient (NP) is received by a hospital to when that patient is added to the specialist list; the average WT for all patients on the list; and time to treatment (TTT), measured from GP referral to treatment. We also used three variables to measure the number of patients waiting: number of NP waiting, number of RP waiting for follow-up, and total number of patients waiting (TPW) as the sum of NP and RP. When we started our project, we disclosed a hitherto unknown problem with a large number of RP that had not been followed up according to their individual schedule (delayed RP), and capacity for treatment of this group became an unanticipated challenge in our work.

As a proxy for planning horizon, we used percent of TPW who were booked for an appointment. For activity data during the interventions we measured the total number of consultations as well as consultations in various diagnostic subgroups and procedures. These data were sufficiently detailed to allow analysis on an hourly basis for the purposes of later booking and planning. We used weekly data from each clinic for waiting and activity parameters, which were analyzed in weekly follow-up with the clinics. Resource data were sampled monthly from the regional salary system and measured by full time equivalents (FTE) for physicians, nurses, and other personnel in each department. Categorizing these data into more specific groups (e.g., secretarial or other patient-related personnel) was not possible because they were integrated in larger units.

Because the topic with delayed RP had not been identified prior to our work, we did not have access to such data in the 26 control clinics where we did not intervene. Neither did we have access to resource data. Our comparison with the control group is therefore limited to development of the monthly WT and NW.

#### Procedure

Each project started by interviewing the clinical management and identifying staff members and front-line personnel to participate. Rather than spending too much time on LEAN education itself, we focused on methods and tools that could identify and resolve problems in individual clinics. Accordingly, we prioritized a simple approach and common structure that could be adapted for application across different departments.

A basic principle was that no extra resources to resolve current problems should be expected during the project period. We focused on practical adaptation of the tools most relevant to our context that would be easy for clinical staff to implement and that could contribute to resolving actual problems without unnecessary delays.

None of our analyses of WT, demand, or resources revealed any characteristics that explained the elevated WT in the 12 months before the intervention. An unanticipated problem was that in addition to a long wait list among the NP, all clinics had extensive RP backlogs. Based on these characteristics, we approached all clinics with a standard concept of simple methods (Table 1) and a common set of targets to be achieved (Table 2).

**Table 1:** Summary of adapted topics in the initial project phases

Topic	Activity
Understanding of processes/Value stream mapping	Detailed analysis of current processes and identification of topics that generated reduced utilization of resources, waste and delays for personnel and patients. Cross-professional teams.
Application of A3 as a problem-solving tool to prioritize actions and evaluate root causes.	Analyses of suggestions for how to organize and design alternative work flows to improve resource utilization and reduce waste
	Identify current condition and root causes of problems
	Define future targets
	Follow up, countermeasures and experimentation
Introduction of team work during interventions involving front line personnel	Team approach to problem solving to safeguard a shared understanding of problems and their solutions, and to build a culture of continual improvement and learning. Multidisciplinary process improvement teams included staff and management representatives involved in the treatment.
Select small changes to ensure early results	Identity most promising initiatives to ensure improvements as soon as possible and select uncomplicated solutions.
Establish regular meetings between the personnel groups involved	Implement huddle meetings and methods that help relay information to problem solvers and create stable structures for continual improvement. Ad hoc ‘lean teams’ to address specific problems when relevant
Involvement of management	Most important, but most challenging, and which did not succeed in all clinics: Engage management in continual problem solving and avoid senior management choosing quick-fixes instead of analyzing and addressing root causes.

^a^A3 is a LEAN method that provides a simple, strict approach that systematically leads toward problem analysis and solving using a single sheet of A3 paper

**Table 2:** Common targets to be achieved during project

	Topic	Targets and actions
Topics and targets from value stream analyses and activity analyses	Evaluation of time from GP referrals to addition to list and response to patient (within the national 10-day limit).	Average evaluation time < 2 days; none > 10 days. Organize regular resources for evaluation of referrals (no batching) < 65 days.
	Long waiting time.	Plan for booking the longest waiters (top 30 each week).
	Long wait list.	Number on wait list reduced to an appropriate level for attaining steady state of a 65-day wait time.
	High number of delayed follow-up patients.	No delayed follow-up patients.
	Inefficient use of secretarial resources due to patients calling for lacking appointments.	Optimization of secretarial resources.
	Large number of patients rebooked.	Longer booking horizon may reduce rebooking?
	Large number of no-shows.	Longer booking horizon may reduce no-shows?
Resources	Clinic management.	Establish regular huddle meetings with involved personnel to discuss current problems and find solutions.
	Cross-professional teams analyzing problems and suggesting solutions.	
	Temporarily buying extra working time.	Temporary increase in physicians’ extended working hours.
	Exporting delayed controls to other providers.	Eliminate old problems that may not be expected to be solved within ordinary steady state resources.

#### Outpatient appointment strategy

Although the tactical and operational approach to booking patients was considered specifically for each clinic, we developed a template that could be adapted and applied across different specialties. Improved planning based on a clearly defined outpatient booking strategy and booking horizon became a priority target in all clinics, because treatment of NP and WT reduction had to be balanced against the large group of delayed RP with significant clinical needs, a previous unknown problem that our project disclosed.

First, we defined the capacity needed for NP and RP, and defined an appropriate set of medical-based priorities. We considered our clinics as multistage (providing multiple services at contact) and multi-server (multiple doctors providing services) [42]. We usually experimented with various service times based on patient diagnosis, predicted complexity, and equipment-related medical services, differentiated for NP and RP where possible. In appropriate cases, some follow-up patient groups were allocated lower service time than NP of the same category. In some clinics we also considered targeted patient selection from the wait list to optimize patient sequences during appointment days as well as whether some overbooking could be relevant. To establish a controllable strategy for eliminating long wait patients and RP backlog, simple rules such as “booking the top 30” long wait or RP backlog each week were applied.

#### Implementation

All actions to implement new procedures were started following an agreed time schedule and based on root problems identified in the value stream analyses. We focused on targets with small steps which were considered most likely to succeed, and assumed that if we could not measure the process, we could not improve it. We avoided any changes in organizational structures. To ensure that all front-line personnel involved knew what was happening, the leaders ensured that everyone in the clinic was kept adequately informed.

Analyses to measure results were performed weekly and results provided to the relevant teams. There was a continuous focus on discovering new challenges and working out how to assess adjustments. We met several employees who had become rather demoralized by the previous situation, and we quickly realized the importance of communicating rapidly with anyone who was skeptical about the process and might resist change. We carefully chose measurements that were considered relevant to the clinical activities studied and communicated positive results as soon as they were agreed to be reliable.

#### Measures used to assess the development of projects

Although TTT would have been a logical measurement and is a useful measure from a clinical perspective, we anticipated that this measure would increase as clinics started to treat patients having extremely long WT, and thus might appear as a “worsening” of the status. Therefore, we focused on WT and TPW, as these measurements were expected to decline if implemented improvements resulted in approaching the prime goal of 65 days WT.

We used STATA version MP 15 for the analyses. The non-parametric Wilcoxon signed-rank test for related samples (paired) was performed to compare data from the 12 clinics for the project start (baseline), 65 days WT, and 6 months after the intervention. To compare our project clinics with the control clinics, we used a difference-in-differences method [43] to estimate the effect of the intervention on the study variables.

## Results

### Analyses from the 12 months before the intervention

**Fig. 1:**
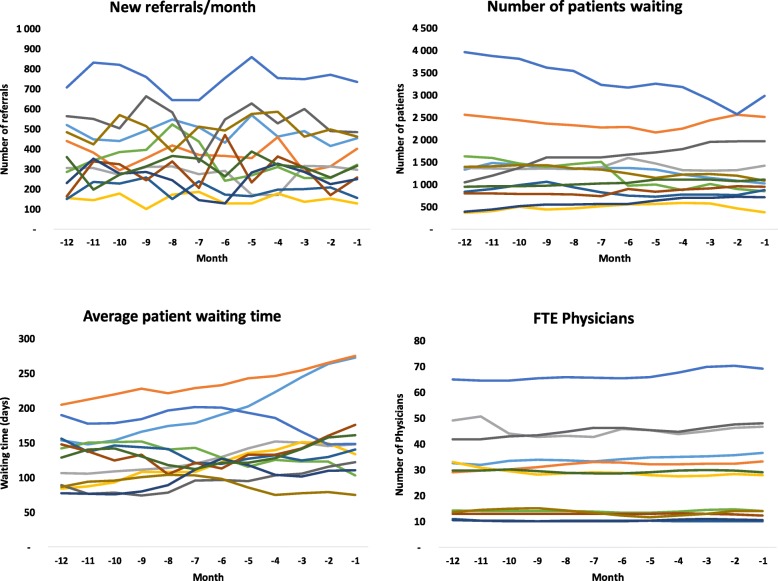
Number of referrals, patients waiting, average waiting time, and full-time equivalent physicians in the 12 months before the intervention. FTE: Full-time equivalent, WT: waiting time

Figure 1 presents the results for the 12 clinics and multiple variables. None of the participating clinics experienced increased demand in this study period, and resources (as measured by physicians and nurses) were constant or slightly increasing. Figure 1 shows that although these variables were fairly constant in the 12 months before the intervention, long WT persisted (and in some clinics increased) despite a declining number of NP waiting.

### Descriptive data from the intervention

**Fig. 2:**
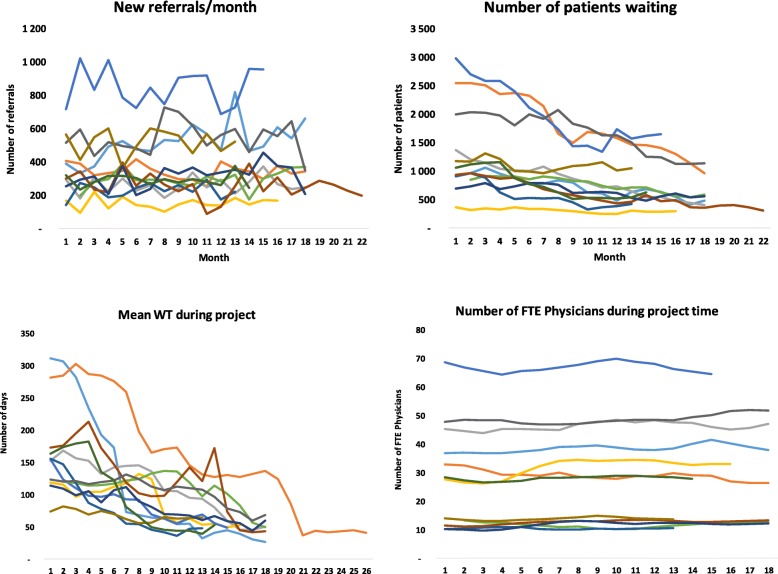
Number of referrals, patients waiting, average waiting time, and full-time equivalent physicians during the project period. FTE: full-time equivalent; WT: waiting time

**Fig. 3:**
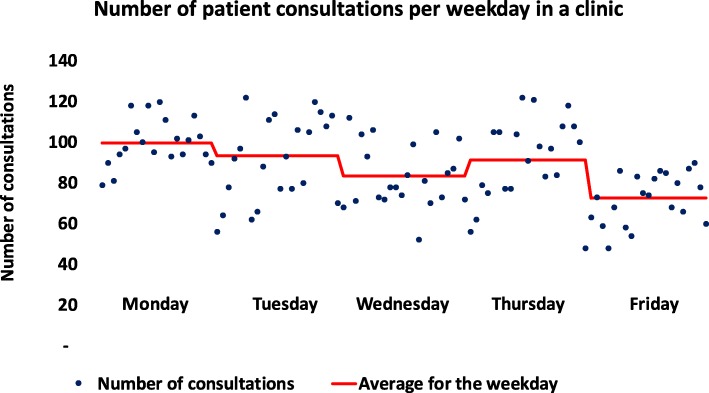
Number of outpatient consultations on weekdays during a 6-month period in one clinic. Legend: The 100% difference between minimum and maximum number of consultations on the same week days and significant lower activity on some week days than others was observed in all clinics. Red line: mean number of consultations each weekday

Our results from the intervention period are presented on an aggregated level because of the large number of variables analyzed across the 12 clinics (Table 3), although some variables are depicted at the clinic level (Figs. 2 and 3). The time to attain a WT of 65 days varied from 4 to 21 months, with the longest projects occurring in clinics with the largest numbers of NW and RP. A total of 19,660 patients were removed from wait/backlog lists (6952 NP and 12,708 delayed RP). The most extreme case was a department with 2800 NP and 3324 RP, and a WT of 312 days. This was reduced to 500 NP waiting and a WT of 37 days at 6 months after the intervention ended. Although Table 3 does not show any significant change in TTT, this variable increased considerably during the first phases of the projects, probably indicating that patients with extreme WT were treated.

**Table 3:** Descriptive data at the start, at 65 days waiting time and at end of project

Variable	Start					By 65 days						End of project					
	Sum	Mean	Min	Max	SD	Sum	Mean	Min	Max	SD	P val *	Sum	Mean	Min	Max	SD	P val#
Waiting time (days)		161	74	312	69		55	37	64	8.3	< 0.001		52	41	74	9.9	< 0.001
Waiting time treated (days)		89	50	124	20		74	50	100	15	< 0.05		60	40	76	12	< 0.05
Evaluation time (days)		5.5	1.9	14	3.8		4.4	2.1	11.9	3.0	ns		3.4	1.1	9.5	2.4	ns
Number of new patients waiting	15,874	1322	369	2980	783	8138	678	248	1450	344	< 0.01	8922	686	296	1650	389	< 0.01
Number of delayed controls	18,700	1558	310	3324	794	8575	714	101	1671	449	< 0.001	5993	461	40	1337	360	< 0.001
Percent of patients booked (%)		27%	11%	63%	17%		63%	29%	93%	20%	< 0.001		61%	36%	91%	17%	< 0.001
Patients waiting/Total activity		280%	136%	581%	141%		126%	65%	226%	55%	< 0.001		85%	0%	201%	53%	< 0.01
Total activity/month	13,525	1127	387	2246	576	14,772	1231	468	2659	671	ns	16,005	1231	508	2804	681	ns
New referrals (number of patients)	4355	362	143	718	168	5149	429	143	916	209	< 0.025	5042	388	168	956	229	ns
Number of physicians (FTE)	346	28	10	68	18	360	30	10	70	19	ns	390	30	10	65	18	ns
Number of nurses (FTE)	557	46	8	150	12	542	45	10	123	12	ns	450	41	11	128	11	ns
Number of consultations/FTE Physician		48	22	107	28		51	19	107	26	< 0.01		54	21	117	28	< 0.05

Note: *FTE* full time equivalents; * *P*-value comparing start and 65 days WT. # *P*-value comparing start and finish. ns = Not significant

Although adding new resources was not an option in our intervention, extra working hours were used in selected clinics for short periods, and some RP who were extremely overdue for follow-up were transferred to other providers. The apparent increase in FTE physicians in the period after 65 days WT resulted from increased staffing in a department that extended its services with a new treatment during the follow up, and one department that was merged with another unit. Apart from minor periodic variations, no other significant changes in staffing were recorded.

Figure 2 shows that the time needed to achieve 65 days WT and the pace of reduction in the number of NPs waiting varied substantially across clinics, and indicates rather stable demand and physician resources during the study period.

In all clinics, there was substantial variation in the daily activity on the same weekdays over time, beyond what could be explained by variation in resources. For example, a two-fold variation in activity on the same weekdays throughout the year unexplained by resource variation, was observed in all clinics. This is depicted for one clinic in Fig. 3. A striking finding was that the planning horizon for future activity was short. In most clinics, only 15–20% of waiting patients had been scheduled for treatment. This booking window was less than 1–2 months ahead and in some cases was only 2–3 weeks, despite pools of NP and RP that in some cases constituted almost 1 year of activity. This represents an extremely short perspective of capacity planning.

**Fig. 4:**
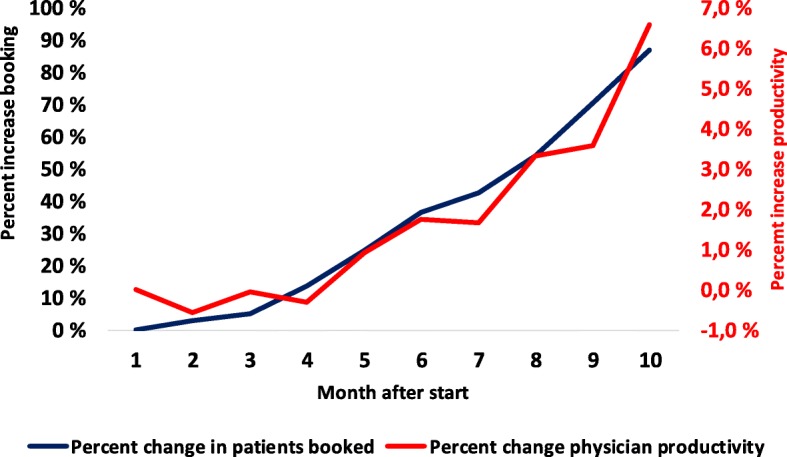
Increase in physician productivity compared with the increase in booking percentage. Legend: Figure show percent in change of average percent booking and change in average physician productivity in 12 clinics during 10 months after project start

We observed an increase in the number of consultations per FTE physician ranging from 4 to 33% in nine of the 12 clinics; two clinics did not change, and one fell by 12%. It increased significantly in the total cohort and the improved physician productivity paralleled an increase in the planning horizon. A naive illustration of this relationship in the total cohort is depicted in Fig. 4 for a 10-month period that included data from all clinics. The percentage increase in physician productivity paralleled an increase in the percentage of patients booked.

### Comparison with non-project clinics

**Fig. 5:**
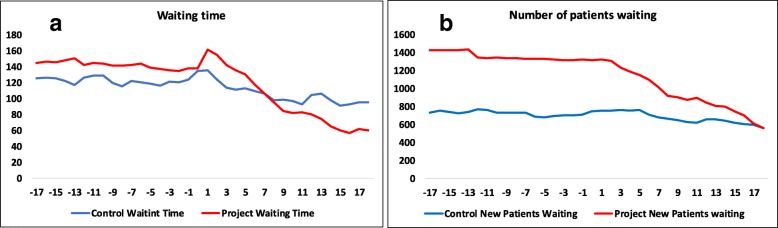
Development of waiting time and number of patients waiting in control and project groups. Legend: Figure shows development of waiting time and number of patients waiting 18 months prior to and up to 18 months after project period

The development of average WT and average NW in our clinics and the control units is shown in Fig. 5. Our clinics had significantly higher starting levels of both WT and NW, and ended up with lower WT than the control clinics. In the difference-in-differences regressions (Table 4), the variable Post Treat Period had a significant effect on the development of both waiting parameters, indicating that our intervention improved the accessibility compared with control clinics.

**Table 4:** Results from the difference-in-differences regression analysis comparing project and control group 18 month before and 18 months after the intervention

Waiting time		(Std. Err.)	*P*-value	Number of Patients Waiting		
	Coefficient			Coefficient	(Std. Err.)	P-value
Post Treatment						
Period						
1	28,18	(10,02)	0,005	12,96	(56,30)	0,818
2	25,09	(10,02)	0,012	1,01	(56,27)	0,986
3	14,11	(10,02)	0,159	−56,41	(56,30)	0,317
4	8,82	(10,03)	0,379	−93,71	(56,32)	0,096
5	1,32	(10,04)	0,896	− 132,46	(56,38)	0,019
6	−15,06	(10,60)	0,155	−165,82	(59,52)	0,005
7	−24,83	(10,73)	0,021	− 211,22	(60,28)	0,000
8	−29,53	(10,75)	0,006	− 269,94	(60,39)	0,000
9	−40,92	(10,90)	0,000	− 301,34	(61,25)	0,000
10	−42,21	(10,92)	0,000	− 331,88	(61,36)	0,000
11	−44,73	(10,94)	0,000	−322,51	(61,44)	0,000
12	−48,74	(11,78)	0,000	− 333,20	(66,15)	0,000
13	−50,62	(12,66)	0,000	− 271,42	(71,14)	0,000
14	−54,43	(12,70)	0,000	− 219,07	(71,36)	0,002
15	−58,85	(12,93)	0,000	− 297,31	(72,61)	0,000
16	−62,90	(13,13)	0,000	−333,16	(73,73)	0,000
17	−65,66	(13,17)	0,000	− 354,65	(73,96)	0,000
18	−69,85	(13,55)	0,000	− 374,09	(76,11)	0,000
Constant	145,50	(20,13)	0,000	1390,87	(113,05)	0,000
Fixed effects for month		Yes			Yes	
Fixed effects for clinics		Yes			Yes	
R^2^		0.57			0.92	
Number of Clinics (Treatment/control)		38(12/26)			38(12/26)	

The use of difference-in difference to identify the intervention effects relies on the parallel trend assumption, that is, that project and control group developed in parallel before the intervention and that the trend for the outcome (waiting parameters) in both groups would have continued to be parallel in the absence of the intervention. The graphs below show development in the project and control group 18 months before and up to 18 months from the start of the intervention.

The results from the difference-in-differences regression analysis (Table 4) show the effect of the intervention on the waiting parameters in the project/intervention group compared with the control group in each post-treatment period. The variable Post Treat Period is an interaction between being in the project/intervention group and receiving an intervention in each post-treatment period (1–18 months).

### Organizational findings

The sessions with value stream analysis revealed several common circumstances across all clinics; unpredictable team communication and unclear procedures between staff, multi-professional autonomy, and hierarchical structures. The front personnel involved in patient treatment often had limited knowledge regarding their mutual tasks, and arenas for regular meetings to follow-up and solve current problems were scarce. This caused bottlenecks, duplicated work, and process delays, resulting in considerable waste, and indicates that organizational factors were important.

The combination of a short planning horizon and a large group of patients who had been waiting for several years without knowing their current status generated numerous patient enquiries which consumed a substantial amount of secretarial resources. In turn, this choked the planning and booking resources.

## Discussion

A major finding of this study was that apparently resistant problems such as extreme WT and RP backlogs in a variety of clinical settings were responsive to relatively simple interventions. Through techniques based on value stream analyses and targeted improvement combined with a focus on planning and increased front personnel involvement, participating clinics were able to reduce WT without adding significant resources. The stable long WT before our intervention was not correlated with increases in demand or obvious changes in resources. Improvements in physician productivity paralleled increases in the planning horizon.

Several organizational issues appeared to be important factors underlying the identified problems. Our findings confirmed that there was a substantial leakage of resources in all clinics due to continuous inquiries from patients seeking lost follow-up appointments or wanted re-bookings. It is not surprising that a planning horizon of a few weeks is not optimal when wait lists correspond to almost 1 year of activity. This problem dropped after the intervention in most clinics.

Despite an observed increase in physician productivity, the large variability in activity on the same weekdays persisted. This is a clear limitation of the impact of the interventions and indicates potential for further improvements. Its not be surprising that the extensive backlogs and large wait lists we observed may crate overload on involved personnel and extend the time needed to obtain gains, and all clinics reported a reduction in overload over time.

Although we derived our methods from LEAN, we did not focus on in debt learning of LEAN as a method as we had to focus on the prime goal of reducing WT within shortest possible time span. The literature suggests that successful LEAN requires a considerable investment in resources, time, and effort [44]. We chose to compromise on the time and resources used for training and theoretical education (important elements in LEAN philosophy and methods), and our approach was more related to focus groups [45]. We did not include steering groups as we considered such functionality to be an important responsibility of clinical management and essential for engagement. Nevertheless, we observed a significant spin-off effect as many of the units started to apply similar methodology beyond the initial focus of our interventions, although there was marked variability in the degree of engagement and sustainability of management involvement and among the different participating actors. A crucial factor was creating a palpable picture of the potential to improve resource use and alleviate stress for front line personnel to facilitate understanding of the usefulness of investing time in the intervention. Our experience was that halfhearted engagement slowed progress, whereas analyses and reports that were targeted to patient accessibility and weekly performance of the units created significant engagement among all personnel groups.

Physicians were the most challenging group to engage because they frequently argued that their time should be prioritized for clinical work. Engagement of physicians may be a crucial factor for several reasons [46]; they are important stakeholders and spokespeople, and have significant influence on various aspects of a clinic’s performance. However, physicians have skills and competence that parallel the core concepts of continuous improvements (as in LEAN). In their everyday patient-related work, physicians’ clinical methods depend on documenting facts and assessment of whether a treatment plan succeeds. This daily documentation is a cornerstone for medical quality and safety in the treatment process, and represents the same basic principles as the core elements of most continuous improvements processes; plan, do, check, and act. This concept has been described by several previous studies. Batalden and Stoltz [47] translated Deming’s “Profound Knowledge” concept into a healthcare context [48]. They pinpointed that traditional improvement driven by intellectual disciplines and professional values may differ from improvement based on knowledge regarding systems, variation, and knowledge theory. We support their idea that quality improvement in healthcare must be based on joined, continuous effort from all healthcare professionals to make changes that will lead to better patient outcomes (health), better system performance (care), and better professional development (learning), with an acknowledgement that this must become an intrinsic part of everyone’s job, every day, and in every part of the system. Improvement thinking should not be considered as a method, but as a workplace culture.

Despite substantial differences among participating clinics, we standardized our methods because we observed several similarities across all units. We were able to adapt our template for all cases, and concluded that similar principles created improvements across different specialties and organizational structures.

### Planning horizon and booking strategy

When we considered how to approach improvements in planning and booking strategies, we drew on literature regarding research on outpatient appointment systems (OAS). Since early pioneering studies by Bailey [49] and Welch [50], there has been growing literature regarding OAS and strategies to balance patient WT and personnel idle time. Previous reviews by Cayirli [42] and Ahmadi-Javid [51] summarized a number of studies from this perspective. However, the majority of relevant papers are based on theoretical and mathematical models that simulate the effects of various strategies. Such theoretical models are challenging to apply in the clinical world, as health personnel may have little background in understanding such complicated relationships. A review by Liu [52] suggested that little is known from real life studies regarding the effect of a booking window on efficiency, no-shows, and out of hospital WT. Tailoring optimal booking strategies depends on the technology used by the institution, and traditional medical patient record systems allow little flexibility to adjust and model different types of booking strategies. As this was also the case our clinics, we prioritized simple strategies that had potential to create immediate effects on our prime targets. Our results provide an example of real life findings in this respect, as we identified large gains from increasing the planning horizon.

Of particular importance in all clinics was the balance between treating new patients versus follow-up for delayed RPs. Most clinics did not regard the RP backlog as a serious problem, possibly because this was not the focus of any political goals and had no economic consequences. Having a large number of delayed RP is unacceptable and the basic principle we used to resolve this problem was similar across all cases. We analyzed and compared the size of the problem and current capacity for each diagnosis group, with capacities continuously adjusted based on medical criteria to balance different patient categories. Of course, the significance of this problem depended on the number of RP compared with the volume of the actual clinic, as a given number in a small clinic may be more serious than the same number in a larger clinic.

### Strengths and weaknesses of this study

We focused our analyses on measurements that clinicians regarded as most clinically relevant. The unsophisticated analyses and methods we used (mostly performed and developed with Microsoft Excel spreadsheets) appeared to be useful across multiple specialties. Much of the OAS literature is based on complex scientific methods and mathematical algorithms that may be unfamiliar to health personnel and therefore have limited impact at the bedside. We believe that clinical monitoring based on simple measures that are directly related the personnel’s everyday work may spark more action among clinicians than more advanced techniques.

Although the participating clinics succeeded in resolving major problems with relatively simple tools, such extreme challenges as we observed may not be encountered in many other contexts. Therefore, care should be taken in generalizing the effect of our methods. The short planning horizon that we observed should also be considered when interpreting the relationship between the booking window and physician productivity. This relationship should also be studied in clinics with less extreme waiting problems.

The LEAN method requires substantial knowledge and practice of its differing elements. We compromised on several LEAN principles to obtain concrete goals in limited time periods. This may jeopardize the long-term results if the studied clinics do not build on their experiences and establish a persisting culture for improvement. Accordingly, we do not know whether our intervention created a culture for continuous improvement beyond our 6-month observation period after the intervention. This will depend on whether the change in culture during our project had power to create continuity among the clinical managers and leaders.

Our study was performed across a variety of specialties and clinical settings. Although this may create potential to generalize some of our findings, there are several factors that need to be considered. We would have preferred to have more detailed resources data, and “consultations” may vary considerably across medical specialties. In some specialties, multiple procedures may be performed by nurses, whereas this is rare in other specialties. However, more detailed data were not available for our study. The participating clinics also faced different challenges; we observed different problems in their processes, and the results showed some variability. Pooling data for a variety of clinics might have resulted in inaccuracies. Nevertheless, our main interpretation that simple methods created substantial improvements in all of the studied clinics is sustained.

We only worked with outpatient clinics. Such clinical activities may closely interact with hospitalized treatment, which might have influenced some of the studied variables of particular resources. Furthermore, several specialties are developing in a way that creates an increasing number of patients in need of chronic, lifelong follow-up. Measuring the volume of such groups is complicated, and these patients may increase demand in a way that might be missed.

Some improvements could be made to the study design. The involvement of physicians should have been more of a focus, as we observed more progress when physicians were engaged. In addition, we should have worked thoroughly across all professions to achieve acceptance that changes were needed. Our experience supported reflections by Bamford and Lodge, who stated that the recognition that change in current operational procedures is needed may often appear late and allow problems to develop too far [29].

## Conclusion

Our study indicates that substantial reductions in WT and waiting lists may be achieved using unsophisticated methods across a variety of medical services, and that the study clinics performed better than a control group. Planning horizons and a focus on capacity are crucial factors. We found no obvious relationship between long WT and demand or resources. Engagement of front line personnel seems important to improve patient availability across differing specialties. Reducing waste and improved use of resources was observed in all participating clinics. Although most clinics showed an increase in physician productivity, the daily activity varied more than the variation in resources. This may indicate potential benefits if the days and periods with the lowest performance are improved to approach the average level in each clinic.

Physicians’ engagement appears to be crucial for success. They should be involved as drivers or champions for such processes by applying the principles and methods from their clinical activities. More research is needed to identify appropriate strategies to achieve physician engagement, and there should be more focus on innovative programs for medical students and resident doctors to integrate improvement skills into professional education and training. Such programs may also be useful for other professions.

## Abbreviations

FTE: Full-time equivalent
NHS: National Health Service (UK)
NP: New patient (patient referred for a first appointment)
RP: Returning patients (patients returning for follow up)
TPW: Total number of patients waiting (includes new and returning patients)
TTT: Time to treatment
WT: Waiting time
